# [^177^Lu]Lu-PSMA-617 (Pluvicto^TM^): The First FDA-Approved Radiotherapeutical for Treatment of Prostate Cancer

**DOI:** 10.3390/ph15101292

**Published:** 2022-10-20

**Authors:** Ute Hennrich, Matthias Eder

**Affiliations:** 1 German Cancer Research Center (DKFZ), Service Unit Radiopharmaceuticals and Preclinical Studies, Im Neuenheimer Feld 280, 69120 Heidelberg, Germany; 2 Division of Radiopharmaceutical Development, German Cancer Consortium (DKTK), Partner Site Freiburg, Freiburg, Germany and German Cancer Research Center, 69120 Heidelberg, Germany; 3 Department of Nuclear Medicine, University Medical Center Freiburg, University of Freiburg, Faculty of Medicine, 79106 Freiburg, Germany

**Keywords:** [^177^Lu]Lu-PSMA-617, Pluvicto^TM^, PSMA, prostate cancer, theranostics, radioligand therapy (RLT)

## Abstract

In March 2022, [^177^Lu]Lu-PSMA-617 (Pluvicto^TM^) was approved by the FDA for the treatment of prostate cancer patients. Until now, the approval has been limited to patients with PSMA-positive metastatic castration-resistant prostate cancer who have previously received other therapy options (such as inhibition of the androgen receptor pathway and taxane-based chemotherapy). [^177^Lu]Lu-PSMA-617, which combines a PSMA-specific peptidomimetic with a therapeutical radionuclide, is used in a radioligand therapy that selectively delivers ionizing radiation to tumor cells, causing their death, while sparing the surrounding healthy tissue. In numerous clinical trials, the efficacy of [^177^Lu]Lu-PSMA-617 was demonstrated.

## 1. Introduction

The radiolabeled drug [^177^Lu]Lu-PSMA-617 (^177^Lu-vipivotide tetraxetan, Pluvicto^TM^, Advanced Accelerator Applications USA, Inc. (AAA, a Novartis company; Millburn, NJ, USA)) has been approved in the US for the treatment of metastatic prostate cancer as of March 2022; more recently, marketing authorization for the drug was granted in Great Britain in August 2022. PSMA-617 is a highly specific drug that targets PSMA (prostate-specific membrane antigen) overexpressed in the prostate tumor tissue. As PSMA is barely expressed on non-prostatic tissue, it has a very low background accumulation in healthy tissue avoiding severe side effects and rendering this therapy safe with low toxicity. In comparison to chemotherapy, PSMA-617 is highly specific for the disease and limits any damage to surrounding tissue.

Pluvicto^TM^ is marketed by Novartis AG (Basel, Switzerland). PSMA-617 was originally developed by the German Cancer Research Center and University Hospital Heidelberg, Germany. According to the FDA approval, the drug is intended for patients who have received previous chemotherapy and who no longer respond to hormone deprivation. In the final phase III trial VISION, [^177^Lu]Lu-PSMA-617 in combination with the standard therapy reduced the overall mortality by 38% and disease progression by 60% [1]. The FDA approval can be considered as a successful final step of the translational research that started in the late 1980s with the discovery of the prostate-specific membrane antigen (PSMA) as a potential target for prostate cancer [2]. Many PSMA-targeting agents have been suggested in the meantime, mainly for the application of imaging prostate cancer. Examples are the FDA-approved diagnostic antibody ^111^In-capromab pendetide (ProstaScint^®^, Cytogen Corporation, Princeton, NJ, USA) [3], the FDA-approved [^68^Ga]Ga-PSMA-11 [4], or several radiolabeled small-molecule inhibitors of PSMA, pioneered by Pomper’s group in the early 2000s [5,6].

**Figure 1:**
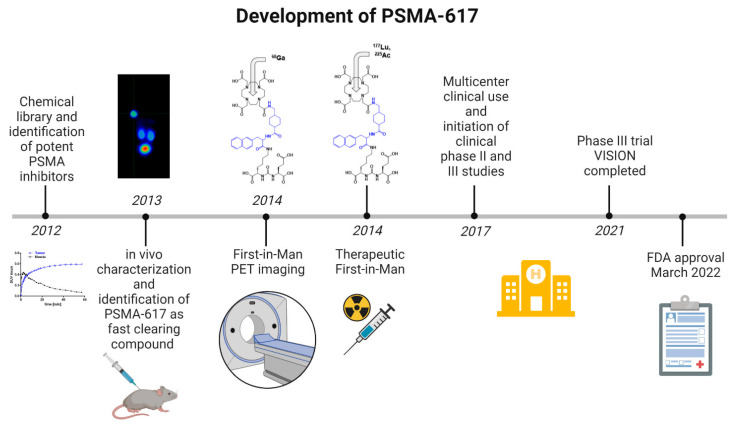
Development of PSMA-617.

The development of PSMA-617 began in 2012 (Figure 1) and is based in part on the earlier discoveries related to the Glu-urea-Lys binding motif. Urea-based PSMA inhibitors were introduced and characterized 20 years ago [7], and the first preclinical development was reported in 2005 [6,8]. Despite good tumor targeting properties, the urea-based low-molecular weight inhibitors of PSMA typically showed high uptake in several organs such as the kidneys, resulting in unfavorable pharmacokinetic properties. Moreover, the tumor retention and related internalization is strongly dependent on structural features not located within the original binding motif. As a consequence, in order to develop a compound suitable for theranostic use, the binding and pharmacokinetic properties had to be optimized with a special emphasis on kidney and tumor uptake. Based on the initial binding motif Glu-urea-Lys, a library of linkers was designed and analyzed in vitro and in vivo [9]. The linker region, i.e., the amino acid sequence located between the binding motif and the radiometal chelator DOTA, was demonstrated to be crucial with respect to the internalization and pharmacokinetic properties of the whole molecule. The work of Benešová et al. identified a compound with ideal properties for the theranostic concept. PSMA-617 revealed the lowest uptake in background organs, particularly the kidneys, while still showing a high tumor accumulation with long retention [10]. In 2014, the first convincing clinical experiences using PSMA-617 were obtained in the diagnosis and radioligand therapy of prostate cancer [11,12]. For further development and commercialization, PSMA-617 was subsequently licensed to ABX advanced biochemical compounds GmbH (Radeberg, Germany) in 2014. In 2017, the US company Endocyte Inc. (Indianapolis, Indiana, USA) acquired the licensing rights from ABX. In 2018, Endocyte Inc. was acquired by Novartis AG (Basel, Switzerland). In the meantime, the clinical value has been further demonstrated in multicentric studies and in the phase III VISION trial, leading to the FDA approval of the drug in 2022.

**Figure 2:**
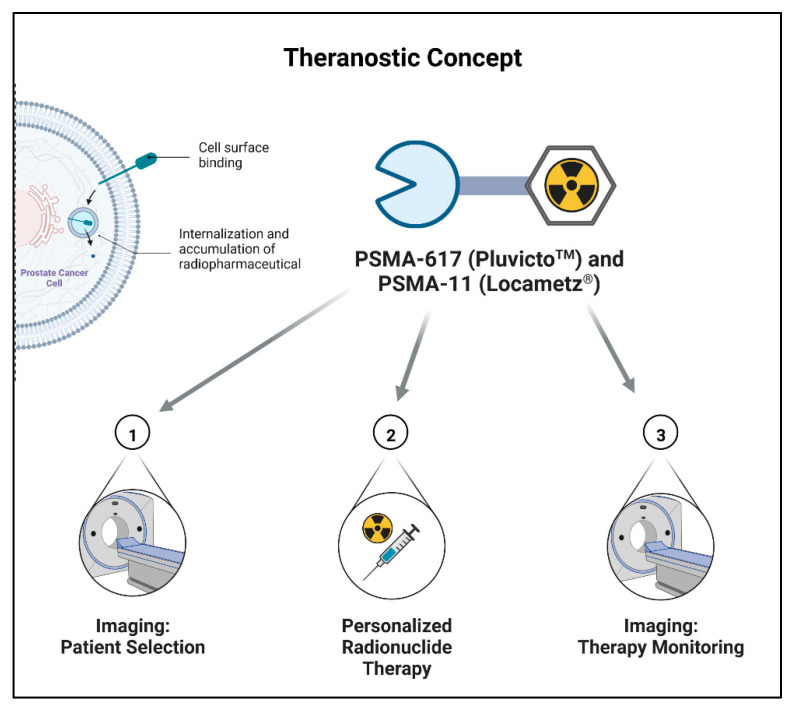
Theranostic concept.

As a radiolabeled drug, PSMA-617 is suitable for use in theranostic approaches of the treatment of cancer patients (Figure 2), which has become ever more important in the last few years with the advance of endoradiotherapy. The term “theranostic” combines both parts of the concept in one word: “thera” for therapy and “nostic” for diagnostic. In a radiopharmaceutical theranostic approach, the patient is first imaged with an appropriate targeted PET (positron emission tomography) or SPECT (single-photon emission computed tomography) tracer, which shows its specific distribution and accumulation at the target site, e.g., a certain receptor overexpressed by the tumor type. If the respective tumor and possible metastases show sufficient uptake of the labeled biomarker, the patient can then be selected for endoradiotherapy with a corresponding radiotherapeutical tracer which has the same or a similar structure as the diagnostic tracer. For therapy monitoring, the patient can again be imaged using a PET or SPECT tracer.

**Figure 3:**
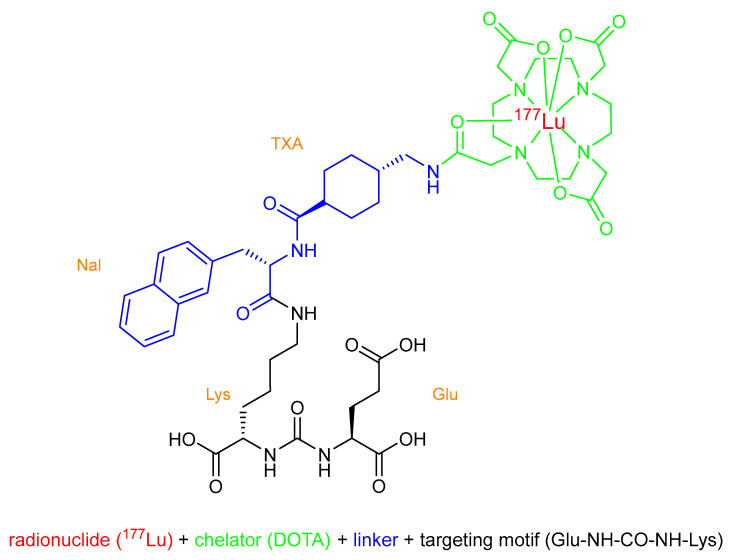
Chemical structure of [^177^Lu]Lu-PSMA-617.

The initial diagnostic step of quantifying the binding of the tracer and PSMA expression in the tumor, respectively, ensures the suitability of the patient to be treated with the radiopharmaceutical in a personalized approach. One prominent example of a theranostic pair is [^68^Ga]Ga-DOTA-TOC (SomaKit TOC^®^) or [^68^Ga]Ga-DOTA-TATE (NETSPOT^TM^) for the diagnostics together with [^177^Lu]Lu-DOTA-TATE (Lutathera^®^) for the therapy of neuroendocrine tumors, which has already been approved by the FDA and EMA a few years ago [13,14]. Due to its structure (see Figure 3), PSMA-617 can be labeled either with ^68^Ga or ^177^Lu, providing exactly the same structure for diagnosis and therapy. However, due to its wide application for the PET imaging of prostate cancer, the FDA-approved [^68^Ga]Ga-PSMA-11, which has a similar structure, is preferred, particularly because new kit preparations have been approved by the FDA: illuccix^®^ (Telix Pharmaceuticals, Inc., Fishers, Indiana, USA) in December 2021 [15] and Locametz^®^ (AAA, a Novartis company, Millburn, NJ, USA) simultaneously to Pluvicto^TM^ [16]. These kit preparations allow for the easy production of the imaging tracer in most nuclear medicine departments. For targeted radionuclide therapy, PSMA-617 can be labeled with the β^−^ particle emitter ^177^Lu. This so-called concept of radioligand therapy (RLT) aims to deliver ionizing radiation to tumor cells while sparing healthy tissue. The underlying principle is the chemical linkage of the particle emitter to an antigen recognition molecule (e.g., peptides, peptidomimetics, antigens, or small molecule inhibitors) [17]. After binding to the PSMA receptor, [^177^Lu]Lu-PSMA-617 is internalized into the PSMA positive cells, resulting in a long retention within these cells (Figure 2); the high energy electrons emitted during the decay can selectively induce tissue and DNA damage, leading to cell death [18]. β^−^ emitters are most commonly used for RLT, which have a low linear energy transfer (LET). The LET is the average energy of the particle emitter that is deposited per track unit on its way [17]. The low deposited energy of β^−^ emitters is only able to yield single-strand breaks of the DNA but can perform this over a longer range (depending on the energy of the emitted electron), which makes them especially suitable for larger tumors. Getting increasingly interesting for RLT, there are alpha emitters, which have a high LET and lower range (40–100 µm) [19]; therefore, they are more suitable for smaller tumors and micrometastases. However, these radiotherapeuticals still require more in-depth studies. The suitability of [^177^Lu]Lu-PSMA-617 for the treatment of prostate cancer patients was demonstrated in numerous clinical trials, leading to its FDA approval, its marketing authorization in Great Britain, and the soon-expected approval by the EMA for use in Europe.

## 2. Chemical Overview

### 2.1. Names and Chemical Structure of [^177^Lu]Lu-PSMA-617 (Pluvicto^TM^)

The active ingredient of Pluvicto^TM^ is the PSMA-binding ligand shown in Figure 3, which was originally named [^177^Lu]Lu-PSMA-617. The molecule consists of the PSMA-binding motif Glu-NH-CO-NH-Lys to which the chelating moiety DOTA is coupled over a linker containing the amino acid 2-naphthyl-L-alanine (Nal) as well as tranexamic acid (TXA). DOTA (1,4,7,10-tetraazacyclododecane-1,4,7,10-tetraacetic acid) is a bifunctional chelator capable of coordinating different radiometals such as gallium-68, yttrium-90 or lutetium-177. In the case of lutetium-177, the coordination is octadentate [20].

Another name for [^177^Lu]Lu-PSMA-617 is lutetium-177 vipivotide tetraxetan, and its IUPAC name is: [^177^Lu]lutetium 2-[4-[2-[[4-[[(2S)-1-[[(5S)-5-carboxy-5-[[(1S)-1,3-dicarboxypropyl]carba-moylamino]pentyl]amino]-3-naphtalen-2-yl-1-oxopropan-2-yl]-carbamoyl]cyclohexyl]-methylamino]-2-oxoethyl]-4,7,10-tris(carboxylatomethyl)-1,4,7,10-tetraazacyclododec-1-yl]acetate.

In the US, Pluvicto^TM^ is commercially available through Advanced Accelerator Applications USA, Inc. (AAA, a Novartis company; Millburn, NJ, USA).

### 2.2. Lutetium-177

The radionuclide lutetium-177 (^177^Lu) is a β^−^ emitter which decays to 100% by emission of electrons to the stable isotope haffnium-177 (^177^Hf) and has a half-life of 6.64 days [21]. For this decay, the maximal β^−^ energy is 496.8 keV (79.4%), but the average β^−^ energy is only 133.6 keV. Depending on the energy of the β-particle, it has different resulting tissue penetration ranges. In the case of ^177^Lu, the maximal range is 2.2 mm, while the average range is 0.67 mm [22]; this classifies the nuclide as a short-range β-particle emitter. This range enables the particle to penetrate and kill several adjacent peptide receptor positive cells while showing limited effects on the neighboring healthy tissue. In addition to the β-particle, low energy γ rays (e.g., 112.9 keV (6.17%) and 208.4 keV (10.4%) [21]) are emitted during the decay of ^177^Lu, which can be used for SPECT imaging or scintigraphy of patients after the injection of [^177^Lu]Lu-PSMA-617, which allows for dosimetry measurements. This makes ^177^Lu an intrinsic theranostic radiometal [23].

The production of ^177^Lu can be achieved by two different routes, either starting with ^176^Lu (direct route) or ^176^Yb (indirect route), and irradiation of the targets with neutrons in a nuclear reactor [24]. When taking the direct route, starting with stable enriched ^176^Lu, the produced radionuclide will be carrier-added (c.a.) as it is not possible to chemically separate ^176^Lu from ^177^Lu. This results in moderate-to-low specific activities of the radionuclide, which steadily decrease as only the nuclide ^177^Lu is decaying. Another drawback of this method is the formation of the radionuclidic impurity ^177m^Lu during irradiation which has a long half-life of 160 days and may complicate radiation protection and waste management. On the other hand, the advantages of this production route are the small amount of enriched target material needed as well as the simple chemical workup of the irradiated target. When producing ^177^Lu by the indirect route, a highly enriched ^176^Yb target is irradiated with neutrons, forming ^177^Yb (t_1/2_ = 1.92 h); this subsequently decays to ^177^Lu [24]. From the irradiated target, ^177^Lu can be chemically separated to obtain the no-carrier-added (n.c.a.) nuclide with high specific activities. Another advantage is that the radionuclidic impurity ^177m^Lu is not formed using this production route. However, a drawback of this method is the high amount of enriched ^176^Yb needed (1 g), which results in two problems. First, the separation of ^177^Lu from the target has to be very effective. Second, ^176^Yb is so far only available from low throughput facilities in Russia [24]. The rising demands of ^177^Lu and current political situation with Russia may result in severely rising prices or even no availability of n.c.a. ^177^Lu at all.

For the production of Pluvicto^TM^, n.c.a. ^177^Lu is preferred, but the use of c.a. ^177^Lu for labeling has also been approved by the FDA. In this case, it is specified on the batch release certificate as different waste management is required [22].

### 2.3. Production and Quality Control of [^177^Lu]Lu-PSMA-617

The production of commercially available [^177^Lu]Lu-PSMA-617 is conducted under full GMP (Good Manufacturing Practice) compliance using dedicated clean rooms and production equipment. To achieve this, the synthesis should be highly/fully automated in suitable synthesis modules. For the preparation and formulation of [^177^Lu]Lu-PSMA-617, different procedures are described in the literature. Depending on the radionuclide used, either of moderate (c.a.) or high specific activity (n.c.a.), the production process may differ.

The use of c.a. ^177^Lu for labeling was extensively studied by the group of Dash et al. with regard to parameters such as ligand/metal ratio, reaction time, reaction pH value, and formulation of the product solution [25,26]. Shortly, the labeling precursor (580 nmol) was reacted with c.a. [^177^Lu]LuCl_3_ (ligand/metal ratio ≈ 2) in ammonium acetate solution at a pH value of ≈ 4.5 at 90 °C for 30 min [26]. The reaction solution was purified by C18 solid phase extraction, and the end product was formulated in physiological saline solution, which contained ethanol and gentisic acid for stabilization. The sterile filtered product solution with a radioactivity concentration of 1.11 GBq/mL can be aseptically dispensed into multiple doses. The overall radiochemical yield of the production was >93% with a radiochemical purity >99%. Stored at 4 °C, the doses can be used for at least 4 days (radiochemical purity of 98.8 ± 0.4%). For the quality control of the product, the radiochemical purity was determined using radio-HPLC as well as by measuring the endotoxin content and sterility. The specific activity of the end product was ≈48 GBq/µmol [25]. The production was not fully automated.

The fully automated GMP compliant production of [^177^Lu]Lu-PSMA-617 using n.c.a. ^177^Lu was described by the group of Scott et al. [27]. The labeling precursor (103 nmol) was reacted with n.c.a. [^177^Lu]LuCl_3_ in sodium acetate solution containing gentisic acid at a pH value of 4.5–5 for 15 min at 95 °C. This resulted in quantitative complexation, and further purification of the product was not required. For stabilization purposes, sodium ascorbate and DTPA were added in water, and the end product was sterile filtered. The overall radiochemical yield was 91 ± 4% with a radiochemical purity of 96.6 ± 0.7%. For quality control, parameters such as radiochemical purity (determined through radio-HPLC and radio-ITLC analyses), pH value, endotoxin content, sterility, and radionuclidic purity were analyzed. The specific activity of the product was estimated to be 88 GBq/µmol.

For the production of [^177^Lu]Lu-PSMA-617 by AAA, the following details for the end product are described in the prescribing information [22]: The injection solution is sterile, preservative-free, clear, and colorless to slightly yellow with a radioactivity concentration of 1 GBq/mL. One dose contains 7.4 GBq at the time of administration in a volume of 7.5–12.5 mL and has a shelf life of 5 days from the date and time of the calibration when stored at <30 °C. The formulation contains acetic acid, sodium acetate, gentisic acid, sodium ascorbate, pentetic acid, and water for injection and has a pH value of 4.5–7. Preferably, n.c.a. ^177^Lu is used for production.

## 3. Medicinal and Pharmaceutical Overview

### 3.1. Clinical Indication

The treatment with [^177^Lu]Lu-PSMA-617 is indicated for male adult patients who have prostate-specific membrane antigen (PSMA)-positive metastatic castration-resistant prostate cancer (mCRPC) [22]. Before PRRT treatment, these patients must be pretreated with androgen receptor (AR) pathway inhibition and taxane-based chemotherapy and no longer elicit a response to such therapeuticals. 

### 3.2. Application

To determine the eligibility for a therapy with [^177^Lu]Lu-PSMA-617, the patients must be scanned for PSMA expression in tumor and metastases before starting the treatment. The PET imaging should be conducted with a suitable theranostic PSMA tracer, such as [^68^Ga]Ga-PSMA-11 [22]. The PET tracer [^68^Ga]Ga-PSMA-11 can be produced in the clinic using commercially available kit preparations such as Locametz^®^ (AAA, a Novartis company, Millburn, NJ, USA) or illuccix^®^ (Telix Pharmaceuticals, Inc., Fishers, Indiana, USA) for labeling with ^68^Ga. For patient selection, the uptake of [^68^Ga]Ga-PSMA-11 in tumor lesions has to be compared with the liver uptake [16]. Tumors or metastases are considered PSMA-positive if their uptake is higher than the liver’s uptake. For eligibility, at least one tumor lesion needs to be positive and all lesions larger than a certain size (in organs ≥ 1 cm, lymph nodes ≥ 2.5 cm, bone ≥ 1 cm).

The recommended treatment schedule with Pluvicto^TM^ is described in the prescribing information [22]: one dosage consists of 7.4 GBq [^177^Lu]Lu-PSMA-617 intravenously injected every 6 weeks for up to six doses. When encountering severe adverse reactions such as myelosuppression or renal toxicity, the interval between doses may be extended to up to 10 weeks or the dosage may be reduced by 20%. The treatment must be discontinued if adverse reactions persist for >4 weeks or if further adverse reactions would warrant a further reduction in dosage.

For patient safety, complete blood counts as well as kidney function tests must be performed before and during treatment with Pluvicto^TM^. When identifying serious abnormalities, the treatment schedule must be adapted. During the VISION trial, the other most common adverse reactions were fatigue, dry mouth, nausea, anemia, decreased appetite, and constipation.

### 3.3. Pharmacology, Pharmacokinetics, and Toxicology

The first clinical experiences in Heidelberg, Germany [11,12] and later a German retrospective multicenter analysis have already shown the beneficial organ distribution and tumor uptake of PSMA inhibitors, providing the rationale for further prospective studies. In the following years, the therapeutic efficacy of [^177^Lu]Lu-PSMA-617 was demonstrated in several phase II studies; the studies conducted in Australia and the United States are the most impactful for the field. Hofman et al. were one of the first to publish a comprehensive report on a phase II study confirming the clinical benefit of [^1^⁷⁷Lu]Lu-PSMA-617 therapy. It was shown that the therapy was accompanied with high response rates and obvious clinical improvements, such as a reduction of pain in patients who have initially progressed after conventional therapy [28]. The authors reported that 57% of patients enrolled in the study had PSA reductions of greater than 50%, and significant improvements in quality of life were reported, which rapidly manifested after initiation of the therapy. On the other hand, the most important side effects reported were grade 1 dry mouth, grade 1 and 2 transient nausea, and fatigue. Grade 3 and 4 thrombocytopenia were reported as rare and partly not clearly attributable to the PSMA-617 radionuclide therapy. All reported toxicities could be easily controlled and were mostly self-limiting. The overall results of this study are in good agreement with those reported in previous retrospective studies. In an extension of this study, the group around Hofman reported even higher efficacy, with PSA response rates of at least 50% in 64% of all cases, whereas toxicity was similar to the first results [29].

Based on these results, the same team in Australia has conducted another multicentric prospective clinical trial investigating the efficacy and clinical performance of [^177^Lu]Lu-PSMA-617 in comparison to cabazitaxel chemotherapy [30]. The study suggests that [^177^Lu]Lu-PSMA-617 is a superior alternative, with higher reported PSA response rates and reduced adverse effects. Another fifty-patient study, again after extensive pre-therapy, confirmed the low toxicity at very high response rates. Improved quality of life was also reported. In the case of progressive disease, this new form of therapy proved to be advantageous over other systemic therapies that often show no further effect [29].

The first multicenter prospective US Study on PSMA-617 (RESIST-PC, NCT03042312) compared two different applied activity dose regimens of [^177^Lu]Lu-PSMA-617 therapy. The first results of this initiative were published by the University of California Los Angeles (UCLA) and represent data of a single-site patient cohort with about two years of follow-up. The results showed no differences between the administration of 6 and 7.4 GBq, which was in line with a retrospective study published before [31]. However, the study showed lower PSA response rates compared with the Australian studies cited above. The authors discussed a more intensive patient selection strategy, including the additional imaging with [^18^F]FDG to improve phenotyping and consequently the success rate [32]. Other criteria such as overall survival (OS) or quality of life, with improved pain levels in 67% of the cases, were comparable to earlier studies. In comparison to former studies, the RESIST-PC trial reported similar outcomes in relation to toxicity. The most frequent side effects were dry mouth, fatigue, and nausea, whereas none of these events were severe [33].

The FDA approval of PSMA-617 was mainly based on the results derived from the most advanced phase III trial VISION, which included 831 patients and ran from June 2018 to October 2019 [1]. The VISION trial was designed for regulatory approval, and the defined endpoints therefore reflected previously accepted approvals by the FDA and other regulatory agencies. In a median follow-up period of about 20.9 months, it was shown that targeted radionuclide therapy with [^177^Lu]Lu-PSMA-617 significantly prolonged both radiographic progression-free survival and overall survival compared to standard treatment alone. Quality of life was not affected by side effects, whereas the risk of death was reduced by 38% and imaging-based disease progression decreased by 60%.

In a sub-study of the VISION trial, dosimetry data was gathered in 29 patients and used for the calculation of whole body and organ absorbed doses. Absorbed doses in selected organs are summarized in Table 1 [22,34]. Organs that received the highest absorbed doses were the lacrimal glands, salivary glands, large intestine, kidneys, rectum, and urinary bladder wall.

In the pharmacokinetic analyses, it was shown that after injection, [^177^Lu]Lu-PSMA-617 was distributed to the gastrointestinal tract, liver, lungs, kidneys, heart wall, bone marrow, and salivary glands within 2.5 h [22]. Studies with the ligand PSMA-617 as well as non-radioactive [^175^Lu]Lu-PSMA-617 showed a binding of 60–70% to human plasma. Primarily renal excretion of unbound [^177^Lu]Lu-PSMA-617 is rapid; 4 h after injection (p.i.), 50% of activity was already excreted, and after 12 h p.i. 70% [35]. Clearance of the radiotherapeutical from the body in the first few hours after administration was fast and then slowed down, resulting in the calculated effective half-lives of 1.7 ± 0.8 h and 41.1 ± 9.3 h, respectively. A whole-body half-life of 42 h was also determined in another study with 87 patients [36].

Even though PSMA expression in healthy tissues such as the salivary glands, kidneys, and small intestine is significantly lower than in tumor tissue, it leads to uptake in these tissues, which may result in toxicity [37]. As described above, most of the observed toxicities have been low grade, with xerostomia or dry mouth being the most common adverse reactions in >30% of patients [38]. While this is often reversible and only mild to moderate, it is nonetheless uncomfortable for the patients [37]. The most serious side effect of treatment with [^177^Lu]Lu-PSMA-617 is hematological toxicity [38]. Observed hematological toxicity is most pronounced in patients with extensive bone metastases, as the uptake in these metastases may lead to radiation-induced damage in the neighboring bone marrow. Renal toxicity resulting in grade 3 or 4 kidney injury occurred in 3% of patients during the VISION trial [22]. In a retrospective Australian study, the renal outcome during a longer follow-up time after treatment (median time 8 months) with either [^177^Lu]Lu-PSMA-617 or [^177^Lu]Lu-PSMA-I&T was evaluated at a mild nephrotoxic risk of 4.5% [39]. During therapy with [^177^Lu]Lu-PSMA-617, the so-called “tumor sink effect” was observed in single cases [34]: while the tumor volume decreases, this leads to an increase in the absorbed dose to the kidneys. This is particularly a concern when patients having an initially low tumor volume are treated. Peters et al., however, showed by small lesion dosimetry that the tumor sink effect in low-volume hormone-sensitive metastatic prostate cancer patients was not relevant in terms of organ toxicity [40].

## 4. Perspective

According to the initial clinical data, PSMA-based targeted radioligand therapy shows high efficacy, with a low risk for side effects. This new type of therapy is very well tolerated and capable of prolonging the life of prostate cancer patients while enabling a high quality of life. Hence, this raises the question about the ideal time point to begin this new type of therapy.

The therapy is very well manageable as it is available within a theranostic concept, allowing for imaging prior to therapy to guarantee efficacy by appropriate patient selection, as well as therapy monitoring. Perspectively within this concept, individualized imaging-based dosimetry is feasible and might play a future role in ensuring strong efficacy and low toxicity for each single patient. Due to these favorable prerequisites and characteristics, the radionuclide therapy approach can be considered as safe, and might be considered earlier in the disease course. The recent approval of [^177^Lu]Lu-PSMA-617 follows the VISION criteria where targeted radionuclide therapy is given after androgen deprivation therapy (ADT), including a novel androgen receptor pathway inhibitor such as abiraterone or enzalutamide, and taxane-based chemotherapy. Thus, the majority of current applications are focused on patients representing a rather poor prognostic cohort of men with metastatic castration-resistant prostate cancer progressing after standard treatments. Consequently, there is a strong need to intensify prospective research on the identification of the best placement of the therapy during various stages of prostate cancer. This means that the future perspective should be on trials with a clinical focus on “real-world” cohorts and earlier time points.

In fact, [^177^Lu]Lu-PSMA-617 therapy has already been considered as an earlier treatment concept through the assessment of the efficacy in prechemotherapy (NCT04689828) or castration-sensitive prostate cancer patients (NCT047201579) [41]. An already-conducted retrospective multicenter analysis revealed that chemotherapy-naive prostate cancer patients had a better outcome, with longer OS rates as compared to patients with a history of chemotherapy [42]. In another prospective trial comparing the efficacy and safety of [^177^Lu]Lu-PSMA-617 and docetaxel in patients who had never received a chemotherapy, it was also concluded that the treatment can be considered earlier in the disease course [43]. Similarly, the therapeutic regimens might be further optimized and adapted to new scenarios. This includes identifying mechanisms of treatment resistance to further improve patient outcomes with, e.g., rationally selected combination therapies. Other current clinical analysis focusing on “real-world” patient cohorts treated under everyday routine practice conditions at an academic center confirmed that the therapy is effective, safe, and well-tolerated, and suggested that prospective studies examine the treatment earlier in the disease course [44].

Another challenge is the proper selection of patients with some aspects currently not fully addressed. Hofman et al., for example, applied very strict selection criteria in an expanded study, which explicitly did not allow patients with low PSMA accumulation in the lesions or cases of PSMA-negative lesions accumulating [^18^F]FDG. This raises the question of the optimal selection of patients for this novel form of therapy to guarantee maximum benefit for the patient. This should be well-balanced, as patients with low or inhomogeneous PSMA expression may also benefit and should not be ignored. This is especially so if, for example, combination therapies are also being considered. For example, patients with a lesion SUV of lower than two were excluded from the VISION trial; however, it is not clear if these patients might respond less favorably than those with higher SUV lesions. Imaging and individual patient-based dosimetry are key for the proper selection and treatment of patients, and there are no doubts that optimal imaging before therapy will result in better therapeutic outcomes. Additional imaging biomarkers may also help in the selection and monitoring of patients to maximize the therapeutic benefit. In this context, first studies have already been performed to better predict the outcome of therapy with PSMA-617. Kind et al. [45] for example recommend focusing on PSA changes at about four weeks after the start of therapy to better predict subsequent biochemical and PET-based response and OS. Further work is required to optimize clinical protocols related to the patient selection and therapy monitoring to fully exploit the potential of the theranostic concept.

Another future challenge is the capacity of current nuclear medicine facilities worldwide to meet the demands arising from the upcoming PSMA-617 therapies and the potential future opportunities of the theranostic concept. A German calculation [46] shows that with an approved therapy using PSMA-617, clinics are expected to be at full capacity or are reaching their limits in terms of bed capacity and staff. In particular, the clinical infrastructure represents a bottleneck in the case of a future expansion to additional patients and indications. Hence, it is a major challenge for the whole field of nuclear medicine in the upcoming years to provide a broad and sufficient global treatment infrastructure. The new PSMA therapy can thus cause a fundamental change in the field of nuclear medicine; according to recent study results, combination therapies with other oncological disciplines might be included in a standard therapy plan, requiring specialized clinical infrastructures with strong knowledge in the nuclear medicine treatment of cancer patients.

Prospectively, numerous individual case observations might play a role that report, for example, the effect of the therapy on other therapeutic interventions, or that discuss the use of the therapy in other tumor entities [47,48]. More trials and clinical evaluations will be necessary to determine the full potential of the new radiopharmaceutical in the future. This also includes the use of other radionuclides such as the alpha emitter Ac-225, which showed substantial antitumor effects even after the failure of a ^177^Lu-based therapy [49].

There are many further questions in the story of PSMA-617 besides the issues discussed here, such as the optimal dose of the radiopharmaceutical, optimal frequency of administration, or influence of standard of care therapies on the receptor expression or the radiosensitivity of the tumor when given (just) before or in combination with [^177^Lu]Lu-PSMA-617. Novel phase II and III trials are currently underway, including for example the PSMAfore trial (NCT04689828) and the PSMAddition trial (NCT04720157). Many further studies will be required to exploit the full potential of this novel therapy concept.

## 5. Conclusions

The novel radiopharmaceutical [^177^Lu]Lu-PSMA-617 causes challenges, but also great opportunities for our healthcare systems, particularly for nuclear medicine and urology. PSMA-617 shows high efficacy and low side effects and represents an important next step towards novel theranostics. The therapy can be well monitored and personalized according to imaging biomarkers so that patients can benefit as much as possible. However, there is still significant potential for improvement, mainly in terms of the optimal introduction of the therapy, as the FDA approval is mainly based on the VISION trial that only included patients with extensive prior treatments. Hence, future clinical work is expected to be focused on less pretreated patients and should include further trials with real-world data analyses to optimize the therapy administration schemes, tumor radiation dose delivery, and efficacy; this will allow for us to fully exploit the high potential of PSMA-617 radionuclide therapy.

## Figures and Tables

**Table 1:** Absorbed organ doses of [^177^Lu]Lu-PSMA-617 in selected organs [22,34].

Organ	Absorbed Dose (Gy/GBq) Mean (±SD) *n* = 29	Calculated Absorbed Dose for 6 × 7.4 GBq (Cumulated Activity 44.4 GBq) (Gy) Mean (±SD)
Lacrimal glands	2.100 (±0.470)	92 (±21)
Salivary glands	0.630 (±0.360)	28 (±16)
Left colon	0.580 (±0.140)	26 (±6.0)
Right colon	0.320 (±0.078)	14 (±3.4)
Kidneys	0.430 (±0.160)	19 (±7.3)
Liver	0.090 (±0.044)	4.0 (±2.0)
Red marrow	0.040 (±0.020)	1.5 (±0.9)
Spleen	0.067 (±0.027)	3.0 (±1.2)
Thyroid	0.260 (±0.370)	11 (±16)
Prostate	0.027 (±0.026)	1.2 (±1.1)
Testes	0.023 (±0.025)	1.0 (±1.1)
Rectum	0.560 (±0.140)	25 (±6.2)
Urinary bladder wall	0.320 (±0.025)	14 (±1.1)
Total body	0.037 (±0.027)	1.6 (±1.2)

## Data Availability

Not applicable.
